# Pink urine syndrome in an anuric patient during continuous renal replacement therapy: A case report

**DOI:** 10.1097/MD.0000000000038986

**Published:** 2024-08-02

**Authors:** Marie Julien, David De Saint Gilles, Nicolas Allou

**Affiliations:** a Réanimation Polyvalente, Centre Hospitalier Universitaire Felix Guyon La Réunion, Saint Denis, France; b Soins intensifs néphrologiques et rein aigu, Hôpital Tenon, Paris, France.

**Keywords:** Pink urine, propofol, renal replacement therapy, uric acid metabolism

## Abstract

**Introduction:**

Pink urine syndrome is a rare, poorly understood condition, often prompted by obesity, insulin resistance, and the drug propofol. It is characterized by pink urine or urine sediment and occurs in the absence of a heme or food-based pigment. The pathophysiology of this syndrome is not yet fully understood but is linked to a uric acid metabolism disorder. Pink urine syndrome is less familiar to anesthesiologists than other propofol infusion complications. Our case report aims to highlight this rarely encountered syndrome, whose both diagnosis and therapeutic may be challenging. We have reported the first case of this syndrome evidenced by the change in color of the effluent bag during continuous veno-venous hemofiltration (CVVHF).

**Case presentation:**

A 61-year-old woman was admitted to the intensive care unit following a recovered cardiorespiratory arrest due to ventricular arrhythmia. She was placed in hypothermia, sedated with propofol (300 mg/h), and started on CVVHF for oligo-anuric acute kidney injury associated with severe metabolic acidosis. A few hours after initiation of CVVHF, the effluent bag turned bright pink. Given the pink color of the effluent bag and the hypothesis of propofol-induced pink urine syndrome, propofol was replaced by midazolam. After stopping propofol, the color of effluent bag lightened. Unfortunately, the patient died on the third day of hospitalization due to diffuse cerebral edema.

**Conclusions:**

We report here the first case of pink urine syndrome as revealed by the change in color of the contents of the CVVHF effluent bag in an anuric patient. This syndrome is rare but significant in anesthesia/intensive care settings, where propofol is a frequently used sedative. Knowledge of this syndrome appears to be important to avoid irrelevant additional investigations and to optimize the therapeutic strategy.

## 1. Introduction

Urine discoloration caused by heme pigments or drugs is a significant clinical symptom that concerns and intrigues clinicians as well as patients. Propofol (2,6-diisopropylphenol) is known to be one of the most common anesthetics causing urine discoloration.^[1]^

It can cause white coloration of the urine due to oil-in-water emulsion,^[2]^ and green coloration due to the phenolic metabolites of propofol produced by the liver.^[3]^

Such urine discoloration is benign and self-limiting. Propofol can also cause the rare clinical phenomenon pink urine syndrome, characterized by pink urine or urine sediment.^[1,4]^

We report here the first case of pink urine syndrome evidenced by the change in color of the effluent bag during continuous veno-venous hemofiltration (CVVHF).

## 2. Case report

A 61-year-old woman with a body mass index of 26.5 kg/m² and a medical history of dyslipidemia, hypertrophic heart disease and recent cholecystectomy surgery was admitted to the intensive care unit (ICU) following cardiorespiratory arrest related to ventricular arrhythmia. Cardiorespiratory arrest was reversed after 6 external electric shocks, administration of 8 mg adrenaline and 450 mg amiodarone. No-flow duration was <2 minutes and low-flow duration lasted 45 minutes. On admission to ICU, the patient’s blood pressure was stabilized with 3.75 mg/h of noradrenaline; her neurological examination was unremarkable under sedation with propofol (300 mg/h) and sufentanil. The patient was placed in hypothermia.

Laboratory analyses showed a lactatemia concentration of 10.3 mmol/L, a pH of 7.13, a sodium bicarbonate concentration of 11 mmol/L and acute kidney injury with a creatinine concentration of 171 µmol/L. Metabolic acidosis and oligo-anuria was diagnosed, and renal replacement therapy by CVVHF was initiated (Prismaflex^®^ machine).

**Figure 1.**
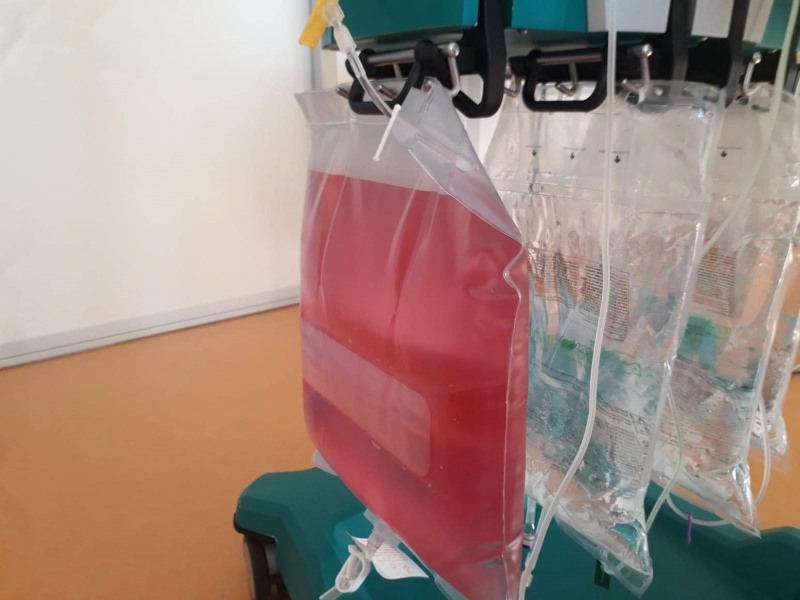
Pink color of the effluent bag during continuous hemofiltration.

A few hours following initiation of CVVHF, the filtrate contained in the effluent bag turned bright pink (Fig. 1). No alarm was triggered by the color change as a false blood leak alarm. The patient did not receive any hydroxocobalamin or other medication that can cause reddish urine discoloration.

A urine dipstick showed 3 + blood, 1 + protein and was negative for glucose, bilirubin, leukoesterase, and nitrite.

**Figure 2.**
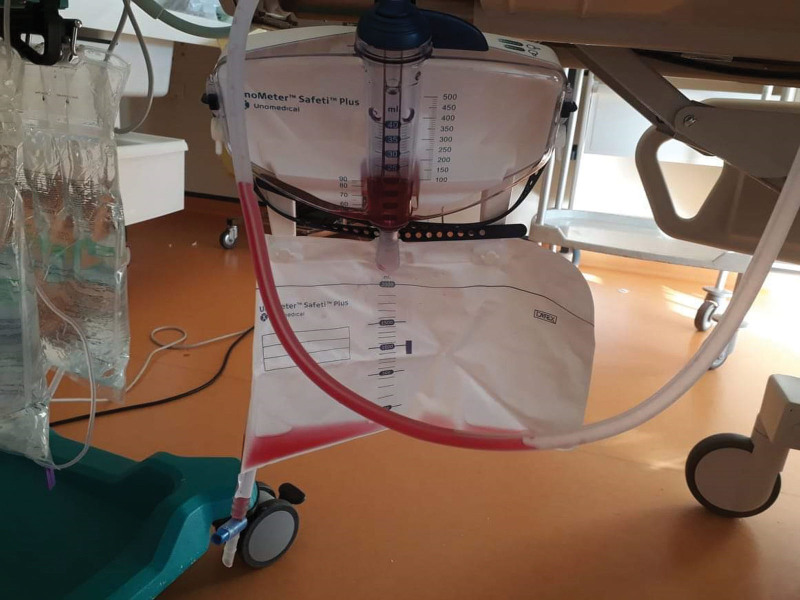
Pink color of the urinary drainage bag.

Urinalysis of the urinary drainage bag (cytobacteriological examination of the urine and urinary sediment analysis) was not possible due to anuria (Fig. 2).

Given the pink color of the effluent bag and the hypothesis of propofol-induced pink urine syndrome, propofol was replaced by midazolam. After stopping propofol, the color of effluent bag lightened.

As a result of diffuse cerebral edema and cerebellar amygdala involvement, the patient’s evolution was unfavorable leading to her death on the third day of hospitalization.

## 3. Discussion and conclusions

Pink urine syndrome is a rare, poorly understood condition, and a diagnostic and etiological challenge. It is characterized by pink urine or urine sediment and occurs in the absence of a heme or food-based pigment. The pathophysiology of this syndrome is not yet fully understood but is linked to a uric acid (UA) metabolism disorder with high urinary UA concentrations in the presence of the drug propofol.

Louis Proust first reported pink urine syndrome in 1800.^[5]^ It was not until almost 200 years later that this syndrome was clinically described in the literature in a surgical cohort of 107 obese patients who had undergone gastric partitioning.^[6]^ The involvement of propofol was only reported in 1996, in 9 Japanese patients presenting with milky pink urine following anesthesia with propofol.^[7]^

By studying and comparing the small number of case reports on pink urine syndrome, it appears to be more often encountered in obese, insulin-resistant male patients with low pH urine, undergoing surgery under propofol sedation.^[1]^ The etiological factor common to all patients is an increase in excretion of urinary UA. The pathophysiology of this syndrome is therefore likely to be linked to UA metabolism disorders, leading to precipitation and crystallization of UA in the urine. UA crystals are normally colorless, but can turn pink if they absorb pink urine pigments.^[8]^ Several factors are known to contribute to UA crystallization. Urine pH has a major influence on the probability of UA precipitation by shifting UA to a protonated, neutral, non-water-soluble form, the origin of crystals.^[1]^ Obesity increases purine biosynthesis and UA production and leads to urine acidification, promoting UA excretion.^[9]^ Low urinary pH and ammoniagenesis defects are commonly observed in insulin-resistant patients, also favoring UA crystallization.^[9,10]^ Moreover, insulin resistance leads to reduced renal urate clearance^[11]^ (via an increase in the urate transporter 1 and a decrease in the adenosine triphosphate-binding cassette efflux transporter G2 (ABCG2)). The activation of vasopressin receptor 1 (e.g. by surgery, stress or low urinary pH) is also involved in pink urine syndrome. Indeed, the release of antidiuretic hormones via these receptors promotes renal UA clearance (downregulation of Glucose transporter 9 and upregulation and crystallization of ABCG2 and sodium-dependent phosphate cotransporter type 1 respectively^[12]^). Environmental temperature also plays a role in urate physiology and pink urine syndrome. When the temperature drops in the operating room or in protective hypothermia post cardiorespiratory arrest, as was the case with our patient, the probability that UA will precipitate increases.^[13]^ Use of propofol increased significantly during the 2020 Coronavirus pandemic to place severely ill patients under deep sedation. It is metabolized primarily by the liver into inactive metabolites such as sulfate and glucuronide. Several case reports have described an association between propofol and pink-colored urine, especially in obese patients.^[1]^ Propofol has both direct and indirect effects on UA metabolism, which may promote pink urine syndrome.^[14]^ It increases urinary excretion of colorless UA and promotes the production of a pink urinary pigment derived from the metabolism of bilirubin by activating the nuclear factor-erythroid 2 related factor 2 (Nrf2)-heme oxygenase-1 antipathway.^[1]^ Nrf2 is also upregulated in situations of oxidative stress. The color of uricin depends on pH and is one of the products of bilirubin metabolism. It appears to be the pigment responsible for the pink color of urine by binding to UA in the presence of an acid pH.^[1]^ Thus, the combination of propofol, obesity, therapeutic hypothermia and cellular stress following cardiorespiratory arrest likely contributed to the development of pink urine syndrome in this patient.

Our study displays several limitations. First, we had no information about the insulin resistance status of the patient, which is a possible pathogenic risk factor of pink urine syndrome. Second, regarding the possible role of uric acid and urinary pH in the pathogenesis of this condition, the values of these 2 parameters were not available in our study. We were also unable to perform microscopic analysis on a sample of CRRT effluent.

To our knowledge, this is the first case report of pink urine syndrome as evidenced by the color of the effluent bag in CVVHF in an anuric patient. This syndrome is rare but significant in anesthesia/intensive care settings, where propofol is a frequently used sedative. The evolution of this syndrome is rapidly favorable after hydration and alkalinization of the urine.^[1,4,14]^ However, we must consider the risk of uric lithiasis in the longer term.^[15]^

## Author contributions

**Conceptualization:** Marie Julien, David De Saint Gilles, Nicolas Allou.

**Methodology:** Marie Julien, Nicolas Allou.

**Validation:** Marie Julien, Nicolas Allou.

**Writing – original draft:** Marie Julien.

**Visualization:** Nicolas Allou.
